# Persistence in time: the hunt for *Bacillus anthracis* at a historic tannery site in Austria reveals genetic diversity thought extinct

**DOI:** 10.1128/aem.01732-24

**Published:** 2025-02-07

**Authors:** Maximilian F. Mayerhofer-Rochel, Florian Himmelbauer, Pierre Reinprecht, Sebastian Herndler, Hugo Weidinger, Hans-Jörg Hellinger, Michael P. Szostak, Gregor Grass, Monika Ehling-Schulz

**Affiliations:** 1 Functional Microbiology, Institute of Microbiology, Center of Pathobiology, Department of Biological Sciences and Pathobiology, University of Veterinary Medicine Vienna 27260 , Vienna, Austria; 2 Armaments and Defence Technology Agency, NBC and Environmental Protection Technology Division, Vienna, Austria; 3 Bundeswehr Institute of Microbiology (IMB) 539152 , Munich, Germany; INRS Armand-Frappier Sante Biotechnologie Research Centre, Laval, Quebec, Canada

**Keywords:** anthrax, *Bacillus anthracis*, soil, Austria, isolation, historic, phylogeography, A.Br.064, reservoir

## Abstract

Identifying and analyzing historic anthrax loci may provide a treasure trove to fill in the gaps of persistence in time and genetic diversity of *Bacillus anthracis*. In countries where anthrax has become a disease of the past, detailed knowledge of the exact location and stability of spores in soil reservoirs is limited. Reviewing archival records may provide valuable clues to unearthing such forgotten sites. Knowledge of anthrax diversity in Austria is scarce, as the only available isolates—originating from the last outbreak in Austria in 1988—cluster in the B.Br.004 (CNEVA) canonical single-nucleotide polymorphism (canSNP) group. Thus, we analyzed archival records on anthrax incidents in Austria to locate historic *B. anthracis* soil reservoirs. In parallel, we tested the performance of different soil processing protocols for the isolation of *B. anthracis* spores to establish a suitable workflow for screening historical anthrax loci. Using an optimized workflow, we were able to isolate viable *B. anthracis* spores 80 years after the last occurrence of anthrax at an abandoned tannery identified through our archival work. Genome analysis of the isolated strains allowed to improve the phylogeographic resolution within the hitherto poorly covered A.Br.064 (V770) canSNP group by linking historical records to genetic information. Furthermore, our results re-emphasize that *B. anthracis* can survive for decades at historic sites and may pose a health threat when such sites are eventually reactivated by climatic factors or human intervention.

**IMPORTANCE:**

*Bacillus anthracis* is a continuing threat from a One Health perspective since it leads to severe infections in animals and humans. Ongoing climate change or human activities can reactivate historical *B. anthracis* loci, previously considered inactive or forgotten. Therefore, knowledge of historic anthrax incidents at abandoned animal processing facilities, such as tanneries or farmyards, along with robust detection protocols, is of prime interest when monitoring this important zoonosis. As shown here, archival records of possible origins of anthrax-contaminated goods received at tanneries are valuable sources and support these efforts. Investigation for viable spores at such historical sites could not only provide new insights into the past genetic diversity and population structure of *B. anthracis* but also provide important information for taking appropriate measures to prevent future outbreaks originating from these sites.

## INTRODUCTION

*Bacillus anthracis*, which causes the zoonotic disease anthrax, is the most notorious member of the *Bacillus cereus sensu lato* group (1). This Gram-positive, endospore-forming bacterium mainly infects ungulate herbivorous mammals during grazing, but other animals, including carnivores, can also contract the disease. Humans are occasionally affected through the handling of infected animal products or by the consumption of undercooked meat (1, 2). After the death of the host animal, vegetative *B. anthracis* cells can be released into the environment through the discharge of body fluids and/or scavengers feeding off the cadaver. The unfavorable growth conditions in the environment and the resulting loss of host-associated CO_2_ trigger sporulation which enables *B. anthracis* to remain dormant as spores for years or even decades (3–6). These endospores may get into contact with new hosts through grazing activities (4), dissemination through wind, heavy rains, landslides, etc. or by human activities such as construction sites, military conflicts, and others (4). Surveillance of *B. anthracis* spores in the environment is of major interest for risk management and possible mitigation. Due to climate change, landslides, and precipitation extremes, the likelihood of the reactivation of historic anthrax loci is expected to increase (5, 7, 8).

In the past two centuries, anthrax was also a common zoonotic disease in central and northern European countries. However, anthrax has become very rare or almost extinct in countries such as Finland, The Netherlands, Germany, Denmark, and Austria (9–15). At the same time, these are the countries for which we have only little information about the domestic *B. anthracis* genotype diversity that typically prevailed in those countries over the past centuries. Most of the information stems from strain collections. There is at least a 150-year history of anthrax contamination in tanneries or wool-sorting factories, as evidenced by the term woolsorters’ disease (16, 17). Previous work has taught us that viable *B. anthracis* isolates can still be recovered from anthrax loci decades later (5, 12, 18–20). For instance, Cherkasskiy (19) provided historical data on Russian anthrax loci from the national anthrax monitoring program, documenting several decades between outbreaks and Brawand et al. (5) described a case in cattle after heavy rainfall, which occurred 40 years after the last case in this region (5). As such, ancient anthrax loci may continue to pose a risk to the environment, animals, and humans. Following the 2001 “Amerithrax” bioterrorist attacks (21, 22) and the increased awareness of *B. anthracis* as a weapon of terrorist or biocrime, research on pathogen detection in environmental samples, and bioforensic traceback analysis (23–27) by genomic studies of isolates worldwide have accelerated. The *B. anthracis* population is characterized by its high clonality, and strains can be assigned to three major lineages: A, B, and C (26, 28). However, the genetic homogeneity of *B. anthracis* makes subtyping and identification of strains sometimes difficult (26). One of the first methods of determining the genetic relationship between strains used various variable-number tandem repeats (VNTR), the so-called multiple-locus VNTR analysis, to distinguish six major genetic groups (28, 29). With the onset of whole-genome sequencing (WGS) and the increase of genome data, the clonality of *B. anthracis* (30–32) and the absence of significant horizontal gene transfer were confirmed (33, 34). Canonical single-nucleotide polymorphisms (canSNPs) were discovered (23, 24, 26, 35, 36). The combination of VNTRs and canSNPs allows the clustering of the *B. anthracis* population into three major clades, called A (A.Br.), B (B.Br.), and C (C.Br.) branch (by VNTRs), subdivided into 13 classical canSNP groups (26, 35). Clade A comprises isolates from all over the world while clade B groups B.Br.004 and B.Br.008 are primarily found in the central European mountainous regions, in Southern Africa and Siberia, respectively (12, 24, 37). Clade C has so far only been recovered from the USA. During a natural anthrax outbreak or after a deliberate, malicious release of the pathogen, genotyping of canSNPs by PCR-based methods (38, 39) provides a rapid means of strain classification.

Numerous protocols have been proposed for the isolation and detection of *B. anthracis* in soil (12, 40–42), but comparison of their respective performance is generally hampered by the different nature of soil matrices, which exhibit widely varying characteristics. This subsequently hinders the effectiveness of isolation and recovery of *B. anthracis* from natural habitats. Due to biosafety issues and practicability, several *B. anthracis* surrogates, including *B. atrophaeus*, *B. cereus*, and *B. thuringiensis*, have been introduced (43, 44) and used to establish methods for the recovery of spores from environmental samples (45, 46). Transfer of this knowledge to *B. anthracis* has led to the now-established isolation methods that focus on the separation of spores from gross soil debris, followed by bacterial germination and cultivation on semi-selective media for subsequent confirmation of *B. anthracis*-specific marker genes by real-time PCR (47, 48).

Austria had its last anthrax outbreak in Tyrol in 1988, and only three *B. anthracis* isolates (besides an imported “Amerithrax” letter-associated isolate from 2001) have been sequenced from strain collections, all belonging to the B branch, probably being autochthonous to the European Alpine region (12). In this work, we combined archival research and field studies to gain further insight into the history and diversity of *B. anthracis* in Austria. Furthermore, we tested the performances of three soil processing protocols for *B. anthracis* spore recovery to determine the optimal method for further analysis of Austrian soils. Using this combinatorial approach, we successfully identified and recovered viable *B. anthracis* spores from soil samples at a relatively ancient (~100 years) abandoned tannery site in Upper Austria. Four new and unique strains of *B. anthracis* were isolated and subjected to canSNP typing and genomic analysis using WGS data.

## RESULTS

### Soil processing protocols differ in their recovery efficacies of *B. anthracis* from Austrian soil samples

To establish a suitable screening protocol for the isolation of *B. anthracis* spores in soil at historic anthrax loci in Austria, the performance of three commonly used soil processing protocols, designated here as BEYER, GABRI, and SILVESTRI (for details see the material and method section), was compared. Two sterile reference soils from Austria were deliberately contaminated with *B. anthracis* str. Sterne spores (3.3 × 10^2^ in total) and subjected to different soil processing protocols. The average colony-forming unit (CFU) per plate and relative plate recovery (*R*_plate_) were calculated to estimate the number of agar plates needed for successful isolation of *B. anthracis* from soil samples. An overview of the performance of the three protocols for the isolation of *B. anthracis* spores is provided in Table 1. The GABRI protocol yielded the lowest total spore recovery (*R*_total_) for Austrian soil samples (20.7% for field soil and 25.1% for alpine soil), while the SILVESTRI protocol showed the best performance (80.1% for field soil and 55.9% for alpine soil), but was the most time intensive. The BEYER protocol showed a similar spore recovery (72.1% for field soil and 43.3% for alpine soil) as the SILVESTRI protocol, but the overall process was about 30 minutes faster. Since the relative recovery of the BEYER protocol is similar to that of the SILVESTRI protocol but faster to conduct, the BEYER protocol was chosen for the screening of soil samples at historic anthrax loci.

**TABLE 1:** Performance of GABRI, SILVESTRI, and BEYER processing protocols for *B. anthracis* isolation from spore-spiked soil samples^*a*^^,*b*^

			Field soil			Alpine soil		
Method	*t* (min)	*V*_reconstitution_ (mL)	${\bar{CFU}}_{plate}$	*R*_plate_	*R*_total_	${\bar{CFU}}_{plate}$	*R*_plate_	*R*_total_
GABRI	60	10	3.4 ± 1.0	1.0	20.7	4.1 ± 2.5	1.3	25.1
SILVESTRI	110	2	66.0 ± 5.1	20.0	80.1	46.1 ± 13.8	14.0	55.9
BEYER	80	2	59.4 ± 5.0	18.0	72.1	35.7 ± 2.6	10.8	43.3

^*a*^ Five hundred microliters of extracted spore suspensions was plated for each protocol. Number of spiked spores was 330 ± 12 for each replicate.

^*b*^ CFU, recovery per plate (*R*_plate_), and the total recovery (*R*_total_) [%] of Sterne on LB agar after 24 h at 37°C.

### Localization, detection, and isolation of *B. anthracis* from historic anthrax sites

Archive material (“Bericht der Landesregierung,” 27 February 1914) stated that a tannery located in Upper Austria imported contaminated hides in the years 1913–1914 from overseas. Additionally, the register of contaminated sites lists an area of the mud ponds of the tannery as a possible anthrax-contaminated site. The mud ponds were used for storage of the accumulated sludge during the tanning process. Sludge was stored in this manner until the then-novel wastewater treatment system was installed in 1991. The sampled area of the tannery was explicitly stated to be possibly anthrax contaminated since the 1920s and not in use since the mentioned archival record of anthrax. Archive records also mentioned a farmyard in the vicinity, where anthrax-diseased cattle were buried on 27 October 1980. Personal communication with the retired official veterinarian revealed the use of soil from the mud ponds as fertilizer at the farmyard as a possible origin of the anthrax outbreak in 1980.

This tannery has been the second largest tannery in Europe with over 1,200 personnel, providing leathery goods for the then Austrian-Hungarian empire and for the automotive industry in Germany. The company operated from 1830 until its closure in 2013 (personal communication with the current owner of the area). The tannery most likely imported anthrax-contaminated hides both locally and from overseas. To test for the presence of still viable *B. anthracis* spores, we collected soil samples from both the tannery and the adjacent farm. In two samples taken at depths of 150 cm from the ground of the mud ponds, animal hair was found accompanied by a distinct smell of putrification. Soil samples were processed using the BEYER protocol and plated undiluted as well as in serial dilutions on semi-selective BamPLET agar plates. The resulting cell lawn of the incubated plates (undiluted samples) was scratched from the plates and suspended in phosphate-buffered saline (PBS). DNA was subsequently isolated and subjected to anthrax-specific qPCR markers to screen for *B. anthracis*. These qPCR tests revealed positive *B. anthracis* signals in one tannery soil sample at a depth of 150 cm, containing animal hair while the other soil samples of the tannery and the farmyard were tested negative. Next, the plates containing the serial dilution of the qPCR-positive soil sample were examined for the growth characteristics typical of *B. anthracis*. A total of 62 presumptive *B. anthracis* colonies were sub-cultivated and tested for the *B. anthracis*-specific genetic markers *PL3*, *cya*, *capB*, and *dhp61* (data not shown). Four colonies tested positive for all four *B. anthracis* marker genes, while all other tested colonies were negative for all four marker genes. To gain insight into the genetic relativeness of these *B. anthracis* isolates, they were subjected to a detailed genetic analysis.

### Sequencing and phylogenetic placement of the new *B. anthracis* isolates

**Fig 1:**
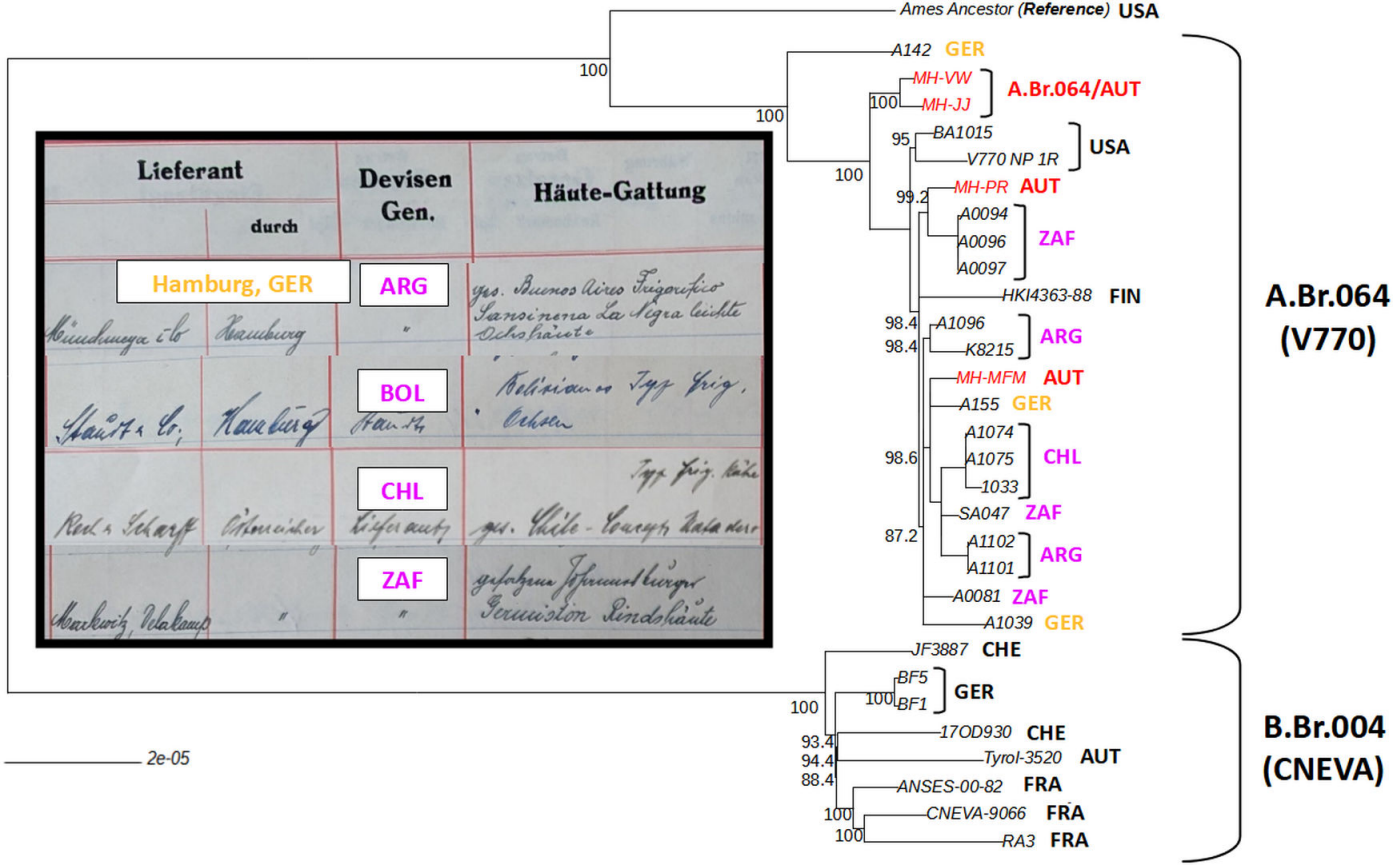
Rooted maximum-likelihood phylogenetic tree including four newly isolated *B. anthracis* strains from a historic anthrax site in Austria. The tree was calculated from chromosomal single-nucleotide polymorphism (SNP) analysis of 2,314 SNP positions (bootstrap confidence from 500 permutations was generated, and the tree with the highest likelihood is shown). The new *B. anthracis* isolates are indicated in red, and all strains originating in South America and South Africa are indicated in magenta. A newly identified sub-branch of A.Br.064/AUT is designated in red. The names of countries of origin are labeled in orange or black. Inset: The pages from an import chart of the tannery receipt book (source: Wolfgang Vogl, Austria; reprinted with permission) show the delivery of hides from South America and South Africa imported through the port of Hamburg (see Table S4).

To determine the respective positions of these novel Austrian *B. anthracis* isolates within the canSNP groups of *B. anthracis*, DNA of the four *B. anthracis* isolates was extracted and subjected to delayed mismatch amplification assay (DMAA) analysis for a first identification and classification of our new *B. anthracis* isolates. Furthermore, the results from DMAA were used to determine possible reference strains from the same geographic origin or canSNP group for sequencing beforehand and to optimize the reference data selection. All four isolates clustered to A.Br.004/A.Br.003 as they exhibited the ancestral genotype for canSNPs C.Br.001 (C-clade), B.Br.003 (B-clade), A.Br.008 (TEA), A.Br.002 (Sterne/Ames), A.Br.003, and A.Br.014 (Aust94) but a derived genotype of A.Br.006 (A-clade) and A.Br.004, hinting at A.Br.064 (V770; Table S1). To confirm the placement of the isolates in A.Br.064 (V770), all four isolates were subsequently subjected to WGS. This analysis resulted in a total of 4.46 GB of reads with an average Q30 of 91.57% and 2.83 GB with 6.51 kb N50 for Illumina and MinION reads, respectively, resulting in a chromosomal coverage of at least >19× for all four *B. anthracis* isolates (Bioproject PRJNA1048328; details of the genome analysis are provided [49]). The analysis of the genome data corroborates the canSNP results of the DMAA analysis. All four isolates were placed into the A.Br.064 (V770) canSNP group (Fig. 1), together with strains from the Americas (Chile, Argentina, Bolivia, and the United States), Africa (South Africa), and Europe (Austria, Germany, and Finland). Furthermore, the analysis of the WGS data revealed that the four isolates from the historical tannery in Austria represent four different genetic lineages. Two of the new Austrian isolates (MH-PR and MH-MFM) fitted well into the shallow phylogeny of the A.Br.064 (V770) canSNP group, while the two others formed a new deeper branching sub-clade. The only strain more basal was a strain isolated from Germany (A142). Notably, A142 and A155 have been isolated from an abandoned tannery site in Germany in 2007 (W. Beyer, personal communication). To facilitate the genotyping of future A.Br.064 isolates, DMAA primers for A.Br.064, as well as the newly designated A.Br.064/AUT sub-branch, were designed (see Table S2).

**Fig 2:**
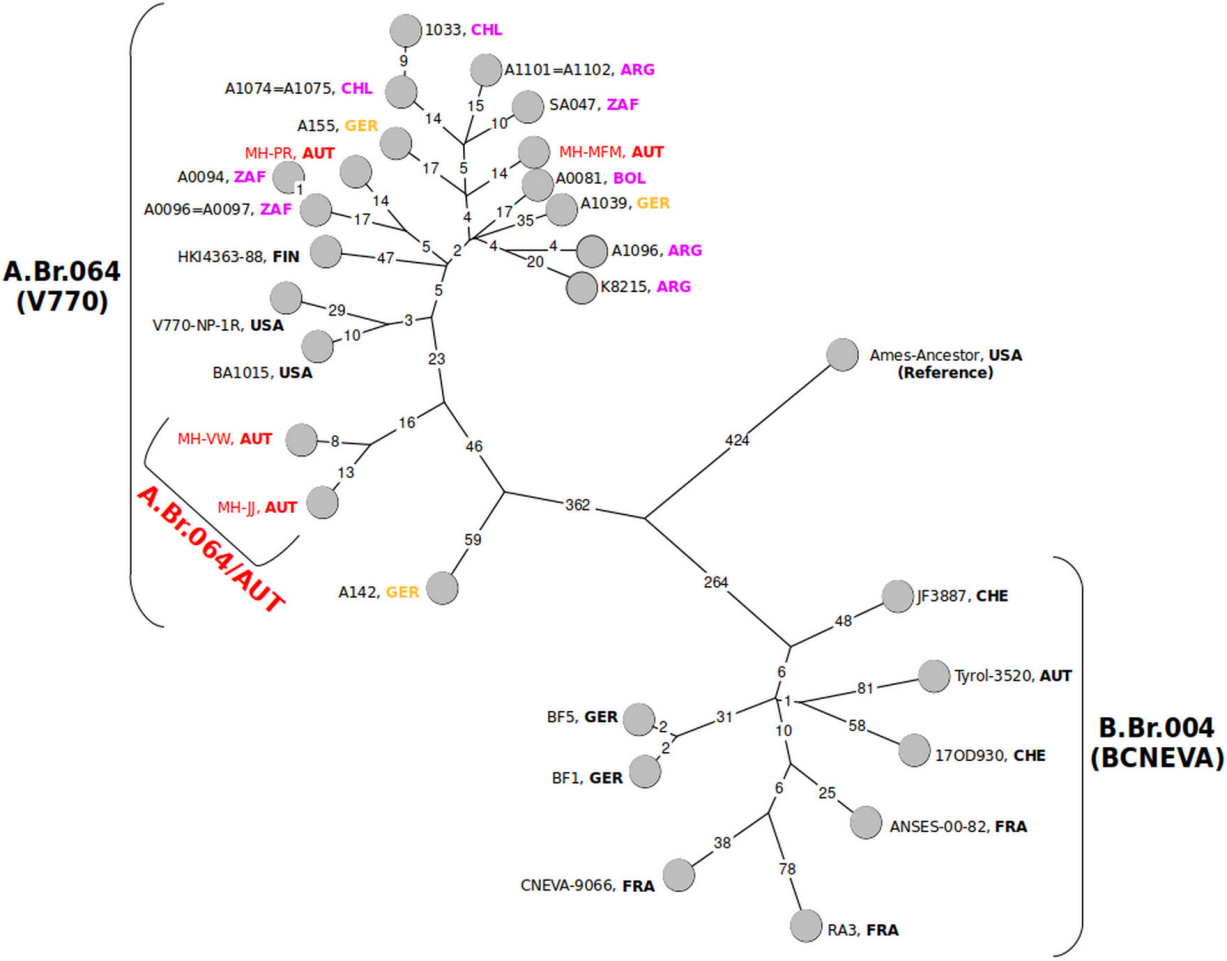
Minimum spanning tree of A.Br.064 (V770) canSNP group and B.Br.004 (CNEVA; with the Ames “Ancestor” reference as root), derived from chromosomal SNPs. Indicated are numerical SNP differences (logarithmic scale) between chromosomes. The geographical origin of strains is indicated using a three-letter country code (e.g., AUT-Austria, FRA-France,…). The new Austrian soil isolates from the historic tannery are highlighted in red. The newly identified sub-branch of A.Br.064, including two out of the four novel isolates from Austria, is designated in red. A.Br.064 group strains originating from Germany are depicted in yellow, and South America and South Africa A.Br.064 group strains are depicted in magenta.

To gain deeper insights into the phylogenetic connections of the new isolates with previously characterized strains, a minimum spanning tree of clade A.Br.064 (V770), B.Br.004 (CNEVA), and the Ames-Ancestor reference genome was calculated (Fig. 2). The genetically closest pair of the new strains (MH-VW and MH-JJ) from the historic tannery, designated as A.B.064/AUT, was separated by 21 single-nucleotide polymorphisms (SNPs), with strain BA1015 from the USA as their closest relative. Strains MH-PR and MH-MFM were 39 SNPs distant from each other and 45 SNPs apart from the node leading to MH-VW and MH-JJ. The closest relatives of strain MH-PR were A0096 and A0094 (South Africa) with 31 and 32 SNPs distance, whereas strain MH-MFM had its closest relatives with A1096 (Argentina) and ZA047 (South Africa) with 26 and 29 SNPs distance, respectively. As future sampling at the historic tannery site may yield additional isolates, the newly designed DMAA primers will aid in the identification of *B. anthracis* strains there and likely also at other historic anthrax sites or outbreaks even possibly in the future.

## DISCUSSION

Challenges arise when sampling historic anthrax loci because one must expect to encounter very low levels of viable spores that can be recovered by cultural methods. Thus, efficient isolation protocols are of utmost importance. Because germination rates of *B. anthracis* spores at ancient anthrax sites (~100 years) may be reduced compared to those at currently active anthrax sites, a high recovery rate is likely necessary for efficient sampling at such sites. To this end, the SILVESTRI and BEYER methods (12, 40, 42) turned out to be the most suitable ones for both soil types tested in the frame of this work. Although the GABRI method has facilitated successful isolation of *B. anthracis* from a comparably relatively recent Italian burial site from the 2000s (41), it may not be the optimal protocol for the analysis of older historic loci when the exact burial site is unknown. For the latter, a higher number of samples is advisable, requiring a method allowing fast and easy processing of samples, such as the BEYER method (12, 40). Using the BEYER method, we were indeed able to isolate viable *B. anthracis* spores from an ancient tannery with a history of anthrax in its vicinity. Such historic anthrax sites may pose a potential threat as they may be reactivated by ongoing climate change, landslides, and extreme weather events. For instance, after more than 20 years without any anthrax outbreaks, an old carcass burial site in an opened cave system flooded by heavy rainfalls caused a bovine anthrax case in Switzerland (5). Furthermore, climate factors leading to the thawing of permafrost have been described as a crucial factor for an anthrax outbreak in Northwest Siberia that affected reindeer as well as humans after 70 years without outbreaks in this region (7, 50).

Additionally, experiments conducted at Scottish Gruinard Island in 1942 during the Second World War contaminated the land, leaving it uninhabitable for over 40 years (51). This deliberate, experimental contamination reflects the high capacity of *B. anthracis* to persist in the environment in its dormant form. It was only after extensive decontamination efforts (52) using formaldehyde that the island of Gruinard was declared “free of anthrax” in 1990 (52). Our results corroborate the findings of these studies that such historical anthrax loci may still contain viable *B. anthracis* spores that persist in time. These may have been long forgotten, but they continue to pose a risk to both animals and human beings.

As revealed by our work, archival records represent a valuable, yet-to-be-exploited source for the discovery of “lost” anthrax loci, even if anthrax is currently unknown in a particular region. Interestingly, the historic receipt book of the tannery investigated in this work stated imports from South America and South Africa in the year 1939 (see Fig. 1 for details). The data contained therein may help to explain the transmission route that led to the isolation of *B. anthracis* strains from the A.Br.064 (V770) clade in Austria. So far, A.Br.064 (V770) has been found predominantly in South America and South Africa (24, 53), while previously isolated Austrian *B. anthracis* from Tyrol belongs to B.Br.004 (CNEVA), which is the predominant genotype in many mountainous European regions (12, 54, 55). Dispersal of *B. anthracis* to other continents is best explained by human activities (e.g., colonization or trade). The diversity of the four Austrian A.Br.064 (V770) clade isolates of this study is a likely result of the tannery site having been contaminated by hides imported from either South America or South Africa. The MH-MFM isolate clustered to South African and South American *B. anthracis* strains (A1096 and SA047), MH-PR was closer to strains originating from South Africa (A0094, A0096, and A0097), and MH-VW and MH-JJ are branching into their own subbranch named A.Br.064/AUT. Of note, the historic tannery receipt book dates only go back to the year 1939, while the archive records list the 1910s–1920s as the first occurrence of *B. anthracis* in this region in Upper Austria. Unfortunately, no older records on imports could be obtained, but trading routes may have already been established in the prior years, as the tannery operated since the 1830s (personal communication, current owner of the tannery area). If our hypothesis on the origin of the new Austrian A.Br.064 isolates (or their ancestors in the contaminated soil) originating from the trade of contaminated hides from South America or South Africa is valid, then these organisms have remained viable in the soil for more than eighty years. However, it remains an open question (56) whether *B. anthracis* persisted in the soil during this time only as dormant spores or if the bacterium underwent active growth cycles in soil (18, 57), resulting in low levels of fresh spores (58). The bifurcated topology of the novel A.Br.064/AUT clade may be the result of germination, vegetative growth, and re-sporulation in the soil or of repeated infection cycles within soilborne organisms as previously suggested (18, 58). Alternatively, spores of these two strains may have been imported from a world region where this genotype is more diverse.

When screening for *B. anthracis* at tannery or animal processing sites, various genotypes covering several major clades may be present in soil samples or other environmental samples such as dust (59, 60). Conversely, in natural outbreak scenarios, typically strains of a single genotype differing only by a few SNPs are usually isolated (13, 18), with rare exceptions (50, 61). If an unexpected anthrax outbreak yields different genotypes, for example after landslides, it could hint at (possibly forgotten) historic anthropogenic artifacts, such as animal (product) processing facilities as well as unregulated or unlawful carcass disposal. Vice versa, it would be prudent to screen *B. anthracis*-positive samples of known but abandoned animal processing facilities for different genotypes of *B. anthracis*, especially if historic import records are available. As thousands of hides have been processed over the decades, sampling expeditions to historic tannery sites may reveal novel genotypes and could provide important insights into the ecology and evolution of *B. anthracis*. To decipher such historical sites, archival records could represent a crucial and valuable resource to consider.

### Conclusion

Prospective outbreak management aiming at estimating the risk potential of historical *B. anthracis* loci should also consider archival records, such as state archives as well as archive material from historic tanneries and animal processing facilities. To the best of our knowledge, this study is the first to utilize state archive material to uncover a historic anthrax locus in Austria, and possibly even Central Europe. Additionally, this study reports the first finding of A.Br.064 (V770) genotypes of *B. anthracis* in Austria. The historic receipt book of the tannery sampled here may explain the spread of A.Br.064 (V770) strains not only into Austria but also into Germany by the trade of hides through the port of Hamburg. Similar events have been proposed for the anthropogenic-driven introduction of isolates of the A.Br.009 group and its progeny to North America from Spain, France, and Italy (62, 63). Therefore, the observed SNP distances between isolates suggest a rather “recent” radiation of the A.Br.064 (V770) clade in human history, possibly within the last few hundred years.

## MATERIALS AND METHODS

### Bacteria and media used in this study

Bacteria were routinely cultivated at 37°C in Lysogeny broth (Luria Bertani, LB) consisting of 1% NaCl, 1% tryptone, and 0.5% yeast extract in deionized water or on LB agar plates supplemented with 1.5% agar (64). For the isolation of *B. anthracis* spores from soil samples, a blood amended modified PLET (BamPLET) agar, consisting of 4.2% PLET agar base (Merck, Darmstadt, Germany) in deionized water supplemented with 2 mg/L trimethoprim (Sigma-Aldrich, St. Louis, MI), 38 mg/L sulfamethoxazole (Sigma-Aldrich, St. Louis, MI), and anthrax selective supplement (Merck, Darmstadt, Germany) consisting of 30,000 U/L polymyxin B and 300,000 U/L lysozyme, 20 mg/L fosfomycin disodium salt (Sigma-Aldrich, St. Louis, MI), 0.03125 mg/L azithromycin dihydrate (Sigma-Aldrich, St. Louis, MI), 0.0625 mg/L erythromycin (Sigma-Aldrich, St. Louis, MI), and 2.5% defibrinated sheep blood, was used. Presumptive *B. anthracis* isolates from soil samples were subcultivated on Standard I Agar (StdI) plates (Carl Roth, Karlsruhe, Germany), which were prepared according to the manufacturer’s instructions.

### Spore preparation of *B. anthracis* str. Sterne

Spores of *B. anthracis* str. Sterne were prepared using Malvar medium (65), which consists of 0.8% nutrient broth Difco (BD, Franklin Lakes, USA) supplemented with 0.05 mM manganese dichloride tetrahydrate (Merck, Darmstadt, Germany), 1 mM magnesium dichloride (Merck, Darmstadt, Germany), and 0.07 mM calcium dichloride (Merck, Darmstadt, Germany). One colony of *B. anthracis* str. Sterne was suspended in 1 mL PBS and heated to 65°C for 30 min to kill vegetative cells. One hundred microliters of this suspension was transferred to 100 mL of Malvar medium (65) in a baffled 500 mL Erlenmeyer flask and incubated at 37°C, 180 revolutions per minute, for 72 h. TWEEN 80 (Sigma-Aldrich, St. Louis, USA) was added to a final concentration of 3%, and incubation was continued for 24 h. The purity of spore suspensions was checked by phase contrast microscopy, and spores were harvested by centrifugation when purity was >95% (66). If purity was less than 95%, spores were washed in Spore wash buffer (SWB) with 3% TWEEN 80 and incubated for another 24 h until purity was sufficiently high. SWB consisted of 8 mM dipotassium hydrophosphate, 2 mM potassium dihydrophosphate (Merck, Darmstadt, Germany), and 0.1% TWEEN 80 (Sigma-Aldrich, St. Louis, USA) in deionized water (67). The spore pellet was washed six times with SWB, subsequently suspended in 40 mL of SWB, and stored at 4°C until use (67). Spores were found to be stable for up to 3 months (for the duration of all experiments; data not shown). Spore preparations were used for the comparison of soil processing protocols and as spike-in for soil samples (see below).

### Comparison of soil processing protocols and estimation of recovery rates with artificially spiked reference soils

Reference soil samples were taken from Lower Austria and in the vicinity of a historic anthrax locus at Upper Austria (180 km distance). Soil samples were dried at 80°C, sieved through a 3 mm sieve, and sterilized at 140°C for 72 h. Sterility was checked by inoculating 1 g of treated soil in 10 mL of LB media after incubation for 24 h at 37°C.

Three different soil spore extraction methods, designated as BEYER (after the first author of the respective publication, Wolfgang Beyer [40]), GABRI (Ground Anthrax *Bacillus* Refined Isolation [41]), and SILVESTRI (after the first author of the respective publication, Erin Silvestri [42]), were used to evaluate extraction efficacy using soils collected in Austria. For reasons of comparability, the same quantity of soil (5 g), the same total numbers of *B. anthracis* str. Sterne spores (3.3 × 10^2^) for spiking the soil samples, the same volume of extraction solution (15 mL), and plating volume (500 µL, V_plating_) for spore re-isolation were used. The recovery quotes of the spores from the artificially contaminated soil samples were determined by plating serial dilutions according to the following protocols.

For the BEYER protocol (12, 40), the soil was added to PBS in a 50 mL screw-cap bottle. After vortexing for 30 min, the suspension was filtered through a sterile gauze inside a sterile syringe to remove larger soil particles. The filtered soil spore slurry was centrifuged at 4,000 × *g* for 15 min. The supernatant was discarded, and the spore pellet was resuspended on 2 mL deionized water (*V*_reconstitution_). The mixture was heated at 65°C for 20 min to inactivate vegetative cells and plated onto LB agar plates. For the GABRI protocol (41), the soil was added to deionized water containing 0.5% TWEEN 20 (molecular grade, Sigma-Aldrich, St. Louis, USA). Samples were vortexed for 30 min. Soil slurries were centrifuged at 657 × *g* for 5 min to pellet large soil particles, and the supernatant was transferred into a new 50 mL tube. Vegetative cells were inactivated by heating the mixture at 64°C for 20 min. A volume of 5 mL of the resulting spore slurry was mixed 1:1 ([vol/vol], *V*_reconstitution_) with tryptose phosphate broth (TPB) amended with 125 µg/mL of fosfomycin. The resulting TPB-Spore slurry was plated onto LB agar plates. For the Silvestri protocol (42), the soil was added to a spore extraction solution (SES) consisting of PBS supplemented with 1% TWEEN 80 and 2% sodium hexametaphosphate. The soil slurry was vortexed for 20 min. After centrifugation at 100 × *g* for 5 min, the supernatant was transferred into a new 50 mL tube. The remaining soil debris was then extracted again using the same amount of SES and shaking, and after centrifugation at 100 × *g*, the supernatant was combined in the same 50 mL tube of the first extraction. The supernatant was then centrifuged at 4,000 × *g* for 30 min to collect the spores. The supernatant was removed, and the spore pellet resuspended in 2 mL deionized water (*V*_reconstitution_) and plated onto LB agar plates.

Each experiment was performed in quintuplicates with three plates each. The LB agar plates from the three different protocols were incubated at 37°C for 24 h. CFU were counted, and recoveries were calculated. The percentage of recovery per plate (*R*_plate_) was calculated by the following formula:


$$R_{plate}={\bar{CFU}}_{replicates}/{\bar{CFU}}_{spikedspores}\times 100$$

The total recovery quota (*R*_total_) was calculated by the following formula:


$$R_{total}={\bar{CFU}}_{replicates}\times \left(\frac{V_{reconstitution}}{V_{plating}}\right)\times 100$$

### Uncovering historic anthrax loci in Austria by archive screening

State archives of Upper Austria were searched for the terms: “Anthrax” and the German term “Milzbrand.” Digital copies of the respective historic documents were retrieved and stored (Oberösterreichisches Landesarchiv; landesarchiv-ooe.at; accessed 2019–01-30). In addition, internal documents from a tannery with a history of anthrax were screened for further information on the origin of potential *B. anthracis* contamination sources.

### Sampling of historic anthrax loci in Austria

Soil samples at the possible anthrax-contaminated historic mud disposal pond sites of the tannery and at the adjacent farmyard, previously identified as historic anthrax loci, were taken by using hand augurs (Ejkelkamp, Giesbeek, The Netherlands). In brief, soil samples from 5, 50, 100, and 150 cm of depth were taken in 500 mL screw-cap containers. Personal protective equipment consisted of DuPont Tyvek 400 protective suites (DuPont, Delaware, USA), FFP3 masks, and chemical-resistant gloves. Before moving to the next digging site, the augur was decontaminated with a 5% terralin PAA (Schülke&Mayr GmbH, Hamburg, Germany) solution. A total of 30 samples were taken at the mud disposal pond and at the farmyard with anthrax history.

### Processing of soil samples from historic anthrax loci, screening, and isolation of *B. anthracis*

Samples were processed according to the BEYER method (see above). For isolation of *B. anthracis*, 500 µL of the resulting slurry was plated on the semi-selective BamPLET agar directly and in serial dilutions. Plates were incubated at 37°C for 24 h. The cell lawn was scraped off the BamPLET agar plates with a sterile loop and resuspended in 500 µL PBS. A 100 µL aliquot was heated to 95°C for 10 min to generate a crude cell lysate. After centrifugation at 4,000 × *g* for 1 min, 5 µL of this cell lawn lysate supernatant was subjected to qPCR analysis. For a broad screening, a triplex qPCR targeting *PL3* (chromosomal marker), *cya* (pXO1 marker), and *capB* (pXO2 marker) genes were used (47). qPCR reactions consisted of 5 µL lysate, 10 µL Luna Universal Mastermix for Probes (New England Biolabs, Ipswich, USA), PCR-grade water, and 1 µL Primer-Probe mix (see Table S2 for further details). qPCR reactions were set by using the MYRA Liquid Handling System (BioMolecular Systems, Australia), cycling comprised initial denaturation for 1 min at 95°C, and 45 cycles of amplification with denaturation for 15 s at 95°C and combined annealing and extension for 35 s at 60°C using the Magnetic Induction Cycler System (BioMolecular Systems, Australia). Data were analyzed using the micPCR v2.12.6 software (BioMolecular Systems, Australia). In addition, the standard chromosomal marker for *B. anthracis dhp61* (48) was used to confirm the presence of *B. anthracis* DNA. Colonies derived from enrichment cultures were screened for morphological features typical for *B. anthracis* (non-hemolytic, ground-glass appearance, and egg-white consistency of colonies) and transferred to StdI agar plates. After incubation at 37°C for 24 h, the cell material of candidate colonies was resuspended in 100 µL of PBS and heated at 95°C for 10 min to lyse the cells and release DNA. After centrifugation at 4,000 × *g*, the supernatant was subjected to triplex qPCR targeting *PL3*, *cya*, and *capB* (47), as well as *dhp61* marker (48). Positive colonies were stored in Microbank (Pro-Lab Diagnostics, Bromborough, UK), at −80°C in a biosafety 3 (BSL-3) laboratory for further analysis.

Four soil samples previously determined negative by the above-mentioned method were deliberately spiked with a total of 2 × 10^2^ and 1 × 10^3^ spores of *B. anthracis* strain Sterne (see above) and used as positive controls for the detection of anthrax-specific qPCR markers in the cell lawn.

### DNA extraction, genome sequencing, and assembly of *B. anthracis* isolates

DNA of *B. anthracis* strains was extracted with the MasterPure Total DNA/RNA Purification kit (Lucigen Middleton, WI 53562, USA) according to the manufacturer with the following modification: a pre-lysis step using 24,839 U of Ready-to-Lyse lysozyme solution (Lucigen Middleton, WI 53562, USA) for 60 min at 37°C was included. WGS was performed on MinION and MiSeq using the SQK-LSK109 chemistry on a R10.4.1 flow cell on the MinION system (Oxford Nanopore Technologies, Oxford, UK) running system software MinKNOW 23.07.8 for the generation of long reads for hybrid assembly and ILMN DNA LP (M) Tagmentation (24 Samples, IPB) for library preparation and the MiniSeq Mid Output Kit (300-cycles) on the MiSeq system (Illumina, California, USA) chemistry as described elsewhere (49). MinION raw reads were first assembled using FLYE v2.9.2-b1786 (68), Illumina raw reads were mapped against the FLYE output using BWA v0.7.17-r1188 (69), and the resulting raw hybrid assembly was further polished using Pilon v1.24 (70) and Ragtag v02.01.00 (71) with a reference guided assembly using the *B. anthracis* str. Ames Ancestor (ASM844v1) reference genome.

### CanSNP typing by delayed mismatch amplification assay

DNA extracts (5 µL) of the *B. anthracis* isolates were subjected to DMAA reactions (38). The two master mixes consisted of either the SNP-specific ancestral or derived primer, the common reverse primer, and 2 × HRM mix (Biozym Biotech Trading GmbH, Vienna, Austria) and water (according to Table S2). Sample and master mix setup was pipetted by the MYRA Liquid Handling System (BioMolecularSystems, Australia) to minimize contamination and pipetting errors. qPCR was performed on magnetic induction cyclers (BioMolecularSystems, Australia) as described above.

### Phylogenetic analysis, SNP discovery, and DMAA primer design

SNP calling was performed using the WGS data of novel isolates with known A.Br.064 and B.Br.004 canSNP-group WGS data and reference genome Ames “Ancestor” (72) from NCBI (Table S1). SNPs were called using parSNP (parameters -C 1000 c -e -u) from HarvestTools (73) v1.3. SNPs were called using all “complete”-flagged genomes from NCBI, in the same fashion as above, as the basis for novel DMAA SNP qPCR assays (Table S2). A maximum-likelihood tree and a minimum spanning tree were calculated using an in-house R script (see Text S1) utilizing R libraries tidyverse (74), phangorn (75), ggtree (76), phytools (77), and devEMF using R v4.3.2 (78). DMAA primers were designed using Geneious Prime 2023.2.1 (https://www.geneious.com) according to previously published methods (38).

## Data Availability

All data in this study are accessible via the NCBI Bioproject PRJNA1048328.

## AFTER EPUB

*[This article was published on 7 February 2025 with Hugo Weidinger's name misspelled in the byline. The byline was updated in the current version, posted on 18 February 2025.]*

## SUPPLEMENTAL MATERIAL

Supplemental text aem.01732-24-s0001.txt R-Script.

Supplemental tables aem.01732-24-s0002.xlsx Tables S1 to S4.

ASM does not own the copyrights to Supplemental Material that may be linked to, or accessed through, an article. The authors have granted ASM a non-exclusive, world-wide license to publish the Supplemental Material files. Please contact the corresponding author directly for reuse.
